# Atherogenic Index of Plasma mediates the association between Life’s Crucial 9 with overactive bladder: a secondary data analysis from NHANES

**DOI:** 10.3389/fendo.2025.1505712

**Published:** 2025-02-28

**Authors:** Hongyang Gong, Xiaomei Lin, Shaoqun Huang

**Affiliations:** ^1^ Department of Oncology Surgery, Fuzhou Hospital of Traditional Chinese Medicine Affiliated to Fujian University of Traditional Chinese Medicine, Fuzhou, Fujian, China; ^2^ Department of Physiology, College of Medicine, Chosun University, Gwangju, Republic of Korea; ^3^ Department of Orthopedics, Fuzhou Hospital of Traditional Chinese Medicine Affiliated to Fujian University of Traditional Chinese Medicine, Fuzhou, Fujian, China

**Keywords:** Life’s Crucial 9, Atherogenic Index of Plasma, overactive bladder, NHANES, mediation analysis

## Abstract

**Background:**

Some studies suggest a potential link between cardiovascular health, lipid, and overactive bladder (OAB). Life’s Crucial 9 (LC9) is a recently developed method for assessing cardiovascular health, while the Atherogenic Index of Plasma (AIP) represents a novel marker of atherosclerotic lipid profiles. However, the relationship between Life’s Crucial 9 and overactive bladder and the role of Atherogenic Index of Plasma in the relationship between Life’s Crucial 9 and overactive bladder is unclear. This study investigates the relationship between Life’s Crucial 9 and overactive bladder and evaluates whether Atherogenic Index of Plasma influences this association.

**Methods:**

This study conducted a cross-sectional analysis of 25,628 U.S. participants in the NHANES database from 2005-2018. Firstly, we used multivariate logistic regression to investigate the relationship between Life’s Crucial 9 and overactive bladder. Subsequently, subgroup analysis and restricted cubic splines (RCS) were further used to verify their relationship. Additionally, mediation analysis was conducted to explore the potential role of Atherogenic Index of Plasma levels in the association between Life’s Crucial 9 and overactive bladder.

**Results:**

A total of 25,628 participants were included in this study, among whom 5,150 reported overactive bladder events. After using multivariate logistic regression to adjust for age, sex, race, marital status, education level, poverty-to-income ratio (PIR), smoking, alcohol consumption, hypertension, diabetes, and hypercholesterolemia, a 10-unit increase in Life’s Crucial 9 was associated with a 28% reduction in overactive bladder incidence (OR = 0.72, 95% CI: 0.69-0.76), while a 1-unit increase in Atherogenic Index of Plasma was associated with a 7% increase in overactive bladder incidence (OR = 1.07, 95% CI: 1.01-1.14). Similar results were obtained when Life’s Crucial 9 and Atherogenic Index of Plasma were categorized into tertiles, with a significant trend (P for trend < 0.05). Restricted cubic spline analysis revealed a linear negative correlation between Life’s Crucial 9 and overactive bladder incidence. Mediation analysis further indicated that 6.49% of the relationship between Life’s Crucial 9 and overactive bladder was mediated by Atherogenic Index of Plasma (P = 0.014).

**Conclusion:**

This study found a significant negative correlation between Life’s Crucial 9 and overactive bladder, with Atherogenic Index of Plasma partially mediating this relationship. These findings highlight the potential link between cardiovascular health and overactive bladder, underscoring the role of Life’s Crucial 9 in reducing overactive bladder incidence, possibly through its effects on lowering lipid levels.

## Introduction

The International Continence Society (ICS) Standardization Committee defines overactive bladder (OAB) as a syndrome characterized primarily by urgency, with or without urgency urinary incontinence, usually accompanied by increased daytime frequency and nocturia, in the absence of obvious pathological changes (1). OAB is a prevalent condition that affects millions of individuals globally, with high prevalence rates in both men and women. This chronic disease significantly reduces the quality of life, leading to social isolation, depression, suicidal mortality (2), and considerable economic burden. In the United States alone, the annual cost of managing OAB is estimated at $82.6 billion (3). As the population ages and living standards improve, the economic burden is expected to increase, underscoring the significant societal impact of this condition. Despite its prevalence and substantial burden, the etiology of OAB remains poorly understood. Identifying modifiable risk factors for OAB is crucial for developing effective prevention strategies and reducing the overall disease burden. Therefore, there is an urgent need to explore potential risk factors and underlying mechanisms to improve the prevention and management of OAB.

Previous studies have established a link between OAB and cardiovascular health, suggesting the possibility of shared pathophysiological mechanisms (4). “Life’s Crucial 9” is a relatively new concept, building on the American Heart Association’s (AHA) earlier “Life’s Simple 7,” which was expanded to include sleep health, forming the “Life’s Essential 8.” More recently, research and opinion articles (5) have proposed the inclusion of mental health, transforming it into “Life’s Crucial 9” (LC9), encompassing nine key indicators: four healthy behaviors (healthy diet, physical activity, smoking cessation, and healthy sleep) and five health factors (weight management, cholesterol control, blood sugar management, blood pressure management, and mental health). LC9 has been shown to strongly predict all-cause and cardiovascular mortality. While these factors have been extensively studied in the context of cardiovascular health, their potential association with OAB remains underexplored. Investigating the relationship between LC9 and OAB could provide a dual benefit for improving both OAB management and cardiovascular health. This not only enriches our understanding of OAB etiology but also offers valuable insights for developing dual prevention strategies.

Lipid metabolism disorders are associated with both cardiovascular disease and OAB. Research suggests that dyslipidemia may play a role in the development of OAB (6). The Atherogenic Index of Plasma (AIP), an indicator that comprehensively reflects lipid metabolism, is an effective tool for predicting cardiovascular risk (7), is strongly linked to oxidative stress (8), chronic inflammation (9), and endothelial dysfunction (10). These factors may contribute to pelvic microvascular dysfunction and exacerbate bladder ischemia-reperfusion injury, ultimately influencing the development and progression of OAB. Therefore, AIP may serve as a mediator in the relationship between LC9 and OAB, reflecting the impact of adverse cardiometabolic conditions on bladder health. Furthermore, AIP provides a potential biological mechanism to quantify this association, offering new insights into the pathophysiology of OAB. Therefore, investigating AIP’s mediating effect in the LC9-OAB association could provide valuable insights into the underlying mechanisms linking cardiovascular risk factors with lower urinary tract symptoms. The use of the National Health and Nutrition Examination Survey (NHANES) database from 2005-2018 offers a unique opportunity to examine this relationship within a large, nationally representative sample. This approach allows for a comprehensive analysis of multiple risk factors and potential mediators while accounting for various confounding variables, thereby enhancing the generalizability and clinical relevance of the findings. A clearer understanding of these relationships could inform the development of novel strategies for preventing and managing OAB in clinical practice.

## Methods

### Study participants

The National Health and Nutrition Examination Survey (NHANES) is an ongoing, stratified, multi-stage sampling program designed to assess the health and nutritional status of adults and children in the United States. It involves a variety of health and nutrition measurements. Each year, NHANES conducts nationally representative surveys on approximately 5,000 individuals, which include both interviews and physical examinations. The interview component covers demographic, socioeconomic, dietary, and health-related information, while the physical examination includes physiological measurements and laboratory tests. Written informed consent is obtained from all participants. The NHANES study protocol is reviewed and approved by the National Center for Health Statistics (NCHS) Research Ethics Review Board.

**Figure 1:**
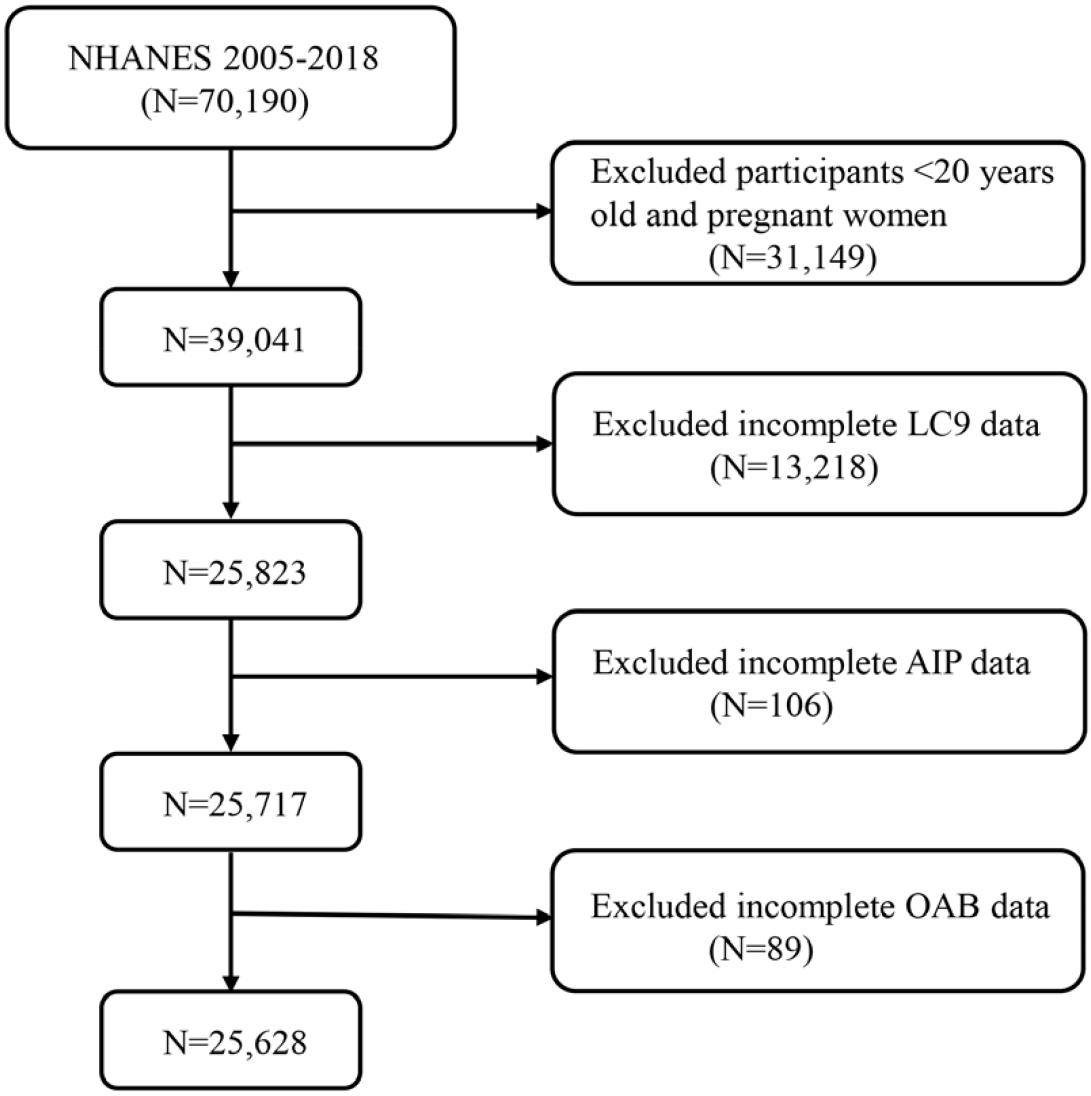
A flow diagram of eligible participant selection in the National Health and Nutrition Examination Survey. LC9, Life’s Crucial 9; OAB, overactive bladder; AIP, Atherogenic Index of plasma.

This study utilized data from seven NHANES cycles spanning 2005 to 2018, applying stringent inclusion criteria to obtain the final study sample. Initially, we excluded 20,190 participants from 70,190 individuals in the original cohort due to being under 20 years old or pregnant. Adolescents were excluded because they are in a developmental stage, which may influence study outcomes, and pregnant women were excluded due to hormonal and physiological changes that could affect the study variables. This step ensured that the study population consisted solely of non-pregnant adults, resulting in a total of 39,041 participants. Subsequently, additional exclusions were made for participants with incomplete data on Life’s Crucial 9 (LC9), Atherogenic Index of Plasma (AIP), and Overactive Bladder (OAB). Specifically, 13,218 participants were excluded due to missing LC9 data, 106 participants due to missing AIP data, and 89 participants due to missing OAB data. After this rigorous selection process, the final sample size included in the study was 25,628 participants (**Figure 1**).

### OAB assessment

According to the definition of OAB, the presence of urgent urinary incontinence and nocturia should be considered indicative of OAB. We assessed urinary incontinence and nocturia using the following three questions from the NHANES KIQ044, KIQ450, and KIQ480 questionnaires (11): (1) In the past 12 months, have you leaked or lost control of a small amount of urine, accompanied by an urge to urinate or pressure, such that you could not reach the bathroom in time? (2) How often did this occur? (3) In the past 30 days, how many times did you typically get up to urinate from the time you went to bed until the time you got up in the morning?

We then used the Overactive Bladder Symptom Score (OABSS) questionnaire to quantify OAB (12). The detailed scoring criteria are listed in **Supplementary Table S4**. Based on previous research (13), the OABSS for each participant was obtained by summing the scores for urgency urinary incontinence and nocturia. In this study, individuals with a total score of ≥3 were considered to be diagnosed with overactive bladder.

### Definition of Life’s Crucial 9

The Life’s Crucial 9 (LC9) framework is a comprehensive health assessment tool (5) that includes nine key indicators: four health behaviors (healthy diet, physical activity, smoking cessation, and healthy sleep) and five health factors (weight management, cholesterol control, blood sugar management, blood pressure management, and mental health). Detailed descriptions of how each participant’s LC9 score was calculated using the NHANES database can be found in **Supplementary Table S1**. Each LC9 indicator is scored on a scale of 0 to 100, with the overall LC9 score derived from the average of the nine individual indicators. This calculation method allows LC9 to provide a holistic health score that reflects an individual’s overall health status. A healthy diet is assessed using the Healthy Eating Index (HEI-2015). The components and scoring criteria of HEI-2015 are detailed in **Supplementary Table S2**. Sleep health, smoking status, physical activity, and mental health are obtained from standardized questionnaires, while BMI, blood pressure, blood sugar, and cholesterol levels are measured by trained professionals using data from the NHANES database (https://www.cdc.gov/nchs/nhanes/index.htm).

### Definition of Atherogenic Index of Plasma

The Atherogenic Index of Plasma (AIP) is an important indicator for assessing cardiovascular disease risk. It is defined by calculating the logarithmic ratio of triglycerides (TG) to high-density lipoprotein cholesterol (HDL-C) in plasma. The specific formula for calculating AIP is as follows: log10[TG(mmol/L)/HDL-C(mmol/L)] (7).

### Covariables

Based on our previous research (14), the study covariates included age, sex, race, marital status, education level, poverty-to-income ratio (PIR), smoking status, alcohol consumption, hypertension, diabetes, and hypercholesterolemia. Details can be found in **Supplementary Table S3**.

### Statistical analysis

To verify the national representativeness of the derived data, sample weights were utilized in all statistical analyses. In our analysis, we computed new weights (2005-2018) as 1/7 × WTMEC2YR, using “WTMEC2YR” as the weighting variable (15). Whereas continuous variables are reported as mean ± standard deviation (SD), categorical variables are displayed as frequencies (percentages). Weighted t-tests were used for continuous variables and weighted chi-square tests were used for categorical data to evaluate differences between the Non-OAB and OAB groups (15). Weighted multivariable logistic regression was used in the study to evaluate the relationship between LC9, AIP, and OAB. Weighted linear regression was used in the study to assess the relationship between LC9 and AIP. To account for confounding factors, we updated the following variables in the regression models (Model 1 was not adjusted for any potential confounders). Model 2 took into consideration factors including age, gender, race, marital status, education level, and poverty status; Model 3 additionally considered factors like smoking, drinking, hypertension, diabetes, and high cholesterol. The results are shown as odds ratios (ORs) or 95% confidence intervals (95% CIs) for β coefficients. Additionally, subgroup analyses were performed to investigate relationships between OAB and LC9 across several categories. Restricted cubic splines (RCS) were used to analyze any nonlinear relationships.

To find out if AIP mediated the effect of LC9 on OAB occurrence, a mediation analysis was carried out based on the preconditions of “statistically significant association between LC9 and AIP” and “statistically significant association between AIP and OAB” (16). The R software’s “mediation” package was used to calculate the mediation effect (15). To verify the robustness of the results, the primary analyses were repeated using multiple imputations by chained equations (MICE). To compute missing LC9, AIP, and OAB data, we employed multiple imputations based on five imputed data sets. All data analyses were performed in R software (version 4.3.1) using the “survey” and “ggplot2” packages for weighted data analysis, constrained cubic spline functions, and visualization. Statistical significance was defined as both sides having a p-value of less than 0.05.

## Result

### Baseline characteristics

This study conducted a comprehensive analysis of 161,119,815 samples, finding an OAB prevalence rate of 16%, closely associated with various demographic and socioeconomic factors. Age and sex were significant factors, with higher OAB prevalence in individuals over 60 years of age and in females. Additionally, higher education levels and non-poverty status were also related to increased OAB risk. The study also found a marked increase in OAB prevalence among individuals with chronic hypertension. Notably, the OAB group had higher levels of AIP and lower levels of LC9 compared to the non-OAB group. For specific details, please refer to **Table 1**.

**Table 1:** Baseline characteristics of all participants were stratified by OAB, weighted.

Characteristic	Overall, N = 161,119,815 (100%)	Non-OAB, N = 135,789,371 (84%)	OAB, N = 25,330,444 (16%)	P Value
**No. of participants in the sample**	25,628	20,478	5,150	-
Age (%)				<0.001
* 20-40*	58,061,358 (36%)	54,327,701 (40%)	3,733,657 (15%)	
* 41-60*	62,398,616 (39%)	53,032,931 (39%)	9,365,685 (37%)	
* >60*	40,659,841 (25%)	28,428,740 (21%)	12,231,102 (48%)	
Gender				<0.001
* Male*	78,471,046 (49%)	68,944,591 (51%)	9,526,455 (38%)	
* Female*	82,648,769 (51%)	66,844,781 (49%)	15,803,989 (62%)	
Race (%)				<0.001
* Non-Hispanic White*	113,731,731 (71%)	96,562,571 (71%)	17,169,159 (68%)	
* Non-Hispanic Black*	16,135,885 (10%)	12,190,188 (9.0%)	3,945,697 (16%)	
* Other*	18,818,242 (12%)	16,268,137 (12%)	2,550,105 (10%)	
* Mexican American*	12,433,957 (7.7%)	10,768,476 (7.9%)	1,665,482 (6.6%)	
Married/live with partner (%)				<0.001
* No*	56,414,900 (35%)	46,320,989 (34%)	10,093,911 (40%)	
* Yes*	104,704,916 (65%)	89,468,383 (66%)	15,236,533 (60%)	
Education level (%)				<0.001
* Below high school*	22,654,588 (14%)	16,928,997 (12%)	5,725,591 (23%)	
* High School or above*	138,465,228 (86%)	118,860,374 (88%)	19,604,853 (77%)	
PIR (%)				<0.001
* Not Poor*	122,351,187 (81%)	105,128,343 (82%)	17,222,844 (74%)	
* poor*	28,928,343 (19%)	22,739,857 (18%)	6,188,486 (26%)	
Smoking (%)				<0.001
* Never*	88,484,965 (55%)	76,304,283 (56%)	12,180,682 (48%)	
* Former*	41,809,121 (26%)	33,866,687 (25%)	7,942,434 (31%)	
* Current*	30,825,729 (19%)	25,618,401 (19%)	5,207,328 (21%)	
Drinking (%)				<0.001
* former*	21,102,280 (13%)	16,025,454 (12%)	5,076,825 (21%)	
* heavy*	32,310,682 (21%)	28,777,953 (22%)	3,532,728 (15%)	
* mild*	59,368,805 (38%)	50,633,829 (38%)	8,734,975 (36%)	
* moderate*	28,052,906 (18%)	24,567,047 (19%)	3,485,859 (14%)	
* never*	16,107,399 (10%)	12,763,070 (9.6%)	3,344,329 (14%)	
Hypertension (%)				<0.001
* No*	99,951,533 (62%)	89,718,127 (66%)	10,233,406 (40%)	
* Yes*	61,168,282 (38%)	46,071,244 (34%)	15,097,038 (60%)	
Diabetes (%)				<0.001
* No*	140,997,532 (88%)	122,081,753 (90%)	18,915,779 (75%)	
* Yes*	20,122,284 (12%)	13,707,619 (10%)	6,414,665 (25%)	
High cholesterol (%)				<0.001
* No*	89,183,788 (62%)	77,420,495 (64%)	11,763,294 (50%)	
* Yes*	54,693,911 (38%)	42,746,027 (36%)	11,947,885 (50%)	
**Mean LC9 score (mean (SD))**	70.63 (13.52)	71.98 (12.98)	63.42 (14.07)	**<0.001**
**Mean HEI-2015 diet score (mean (SD))**	39.40 (31.32)	39.39 (31.35)	39.45 (31.16)	0.867
**Mean physical activity score (mean (SD))**	71.96 (40.97)	74.12 (39.72)	60.40 (45.38)	**<0.001**
**Mean tobacco exposure score (mean (SD))**	71.39 (38.65)	71.91 (38.61)	68.60 (38.76)	**<0.001**
**Mean sleep health score (mean (SD))**	83.48 (24.19)	84.35 (23.38)	78.80 (27.68)	**<0.001**
**Mean psychological health score (mean (SD))**	89.43 (23.06)	91.34 (20.82)	79.18 (30.56)	**<0.001**
**Mean body mass index score (mean (SD))**	60.50 (33.55)	62.31 (33.04)	50.74 (34.60)	**<0.001**
**Mean blood lipid score (mean (SD))**	64.34 (30.26)	64.86 (30.34)	61.58 (29.70)	**<0.001**
**Mean blood glucose score (mean (SD))**	85.94 (24.06)	87.96 (22.40)	75.13 (29.19)	**<0.001**
**Mean blood pressure score (mean (SD))**	69.27 (30.92)	71.58 (30.04)	56.89 (32.59)	**<0.001**
LC9, Tertile (%)				<0.001
* T1*	55,559,802 (34%)	41,421,830 (31%)	14,137,971 (56%)	
* T2*	50,457,961 (31%)	43,766,761 (32%)	6,691,200 (26%)	
* T3*	55,102,053 (34%)	50,600,780 (37%)	4,501,273 (18%)	
**AIP (mean (SD))**	0.05 (0.81)	0.04 (0.81)	0.11 (0.78)	**<0.001**
AIP, Tertile (%)				<0.001
* T1*	53,716,886 (33%)	46,264,670 (34%)	7,452,216 (29%)	
* T2*	53,711,199 (33%)	44,976,541 (33%)	8,734,658 (34%)	
* T3*	53,691,730 (33%)	44,548,160 (33%)	9,143,570 (36%)	

Mean (SD) for continuous variables: the P value was calculated by the weighted Students T-test.

Percentages (weighted N, %) for categorical variables: the P value was calculated by the weighted chi-square test.

LC9, Life’s Crucial 9; OAB, overactive bladder; AIP, Atherogenic Index of plasma; HEI-2015, Healthy Eating Index-2015; PIR, Ratio of family income to poverty.

The bold values are less than 0.05.

### The association between LC9 and OAB

As shown in **Table 2**, we used three different models to assess the relationship between LC9 and OAB. In Model 3, correcting for all variables, each 10-unit increase in LC9 was associated with a 28% reduction in the odds of OAB [0.72 (0.69, 0.76)]. In addition, in Model 3, the odds of an OAB occurring in the third tertile (T3) relative to the first tertile (T1) of LC9 are reduced by 53% [0.47 (0.40, 0.54)]. In addition, from T1 to T3, the ORs decreased progressively with increasing LC9 (P for trend <0.001). The results of Model 1 and Model 2 were also consistent.

**Table 2:** Logistic regression on the association between LC9, AIP, and OAB, NHANES 2005–2018.

Characteristics	Model 1 [OR (95% CI)]	*p-value*	Model 2 [OR (95% CI)]	*p-value*	Model 3 [OR (95% CI)]	*p-value*
LC9 - OAB						
Continuous (per 10 scores)	0.63 (0.61, 0.65)	<0.001	0.70 (0.67, 0.73)	<0.001	0.72 (0.69, 0.76)	<0.001
Tertile						
T1	1 (ref.)		1 (ref.)		1 (ref.)	
T2	0.45 (0.40, 0.50)	<0.001	0.54 (0.48, 0.60)	<0.001	0.61 (0.54, 0.69)	<0.001
T3	0.26 (0.23, 0.29)	<0.001	0.37 (0.33, 0.43)	<0.001	0.47 (0.40, 0.54)	<0.001
*P for trend*	<0.001		<0.001		<0.001	
AIP - OAB						
Continuous	1.12 (1.07, 1.17)	<0.001	1.20 (1.13, 1.28)	<0.001	1.07 (1.01, 1.14)	0.041
Tertile						
T1	1 (ref.)		1 (ref.)		1 (ref.)	
T2	1.21 (1.07, 1.36)	0.002	1.20 (1.06, 1.37)	0.005	1.08 (0.93, 1.24)	0.300
T3	1.27 (1.14, 1.42)	<0.001	1.41 (1.24, 1.60)	<0.001	1.15 (1.00, 1.30)	0.045
*P for trend*	<0.001		<0.001		0.045	

Model 1: no covariates were adjusted.

Model 2: age, gender, education level, marital, PIR, and race were adjusted.

Model 3: age, gender, education level, marital, PIR, race, smoking, drinking, hypertension, diabetes, and high cholesterol were adjusted.

LC9, Life’s Crucial 9; OAB, overactive bladder; AIP, Atherogenic Index of plasma; PIR, Ratio of family income to poverty; OR, odds ratio; CI, confidence interval.

**Figure 2:**
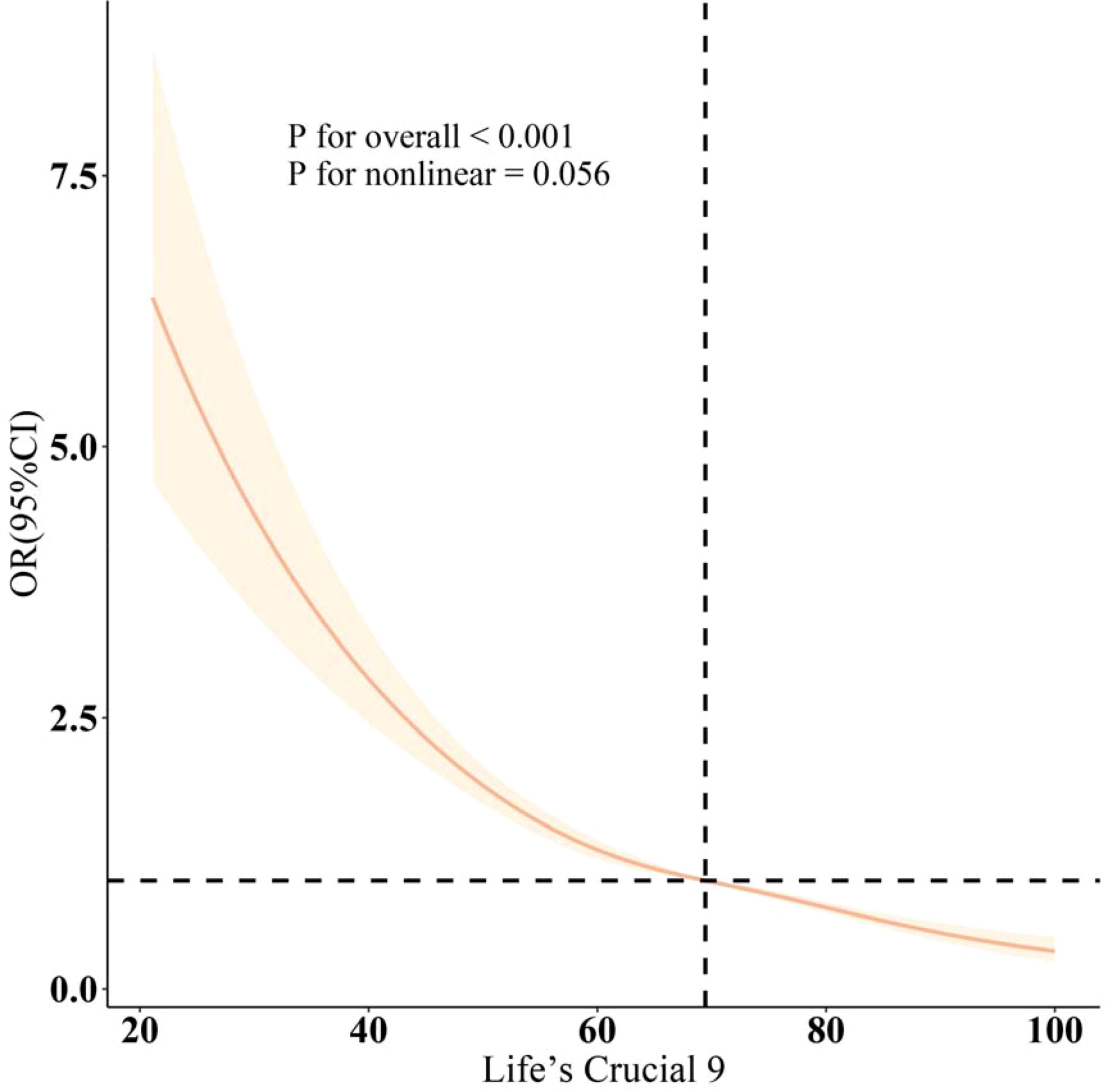
Dose-response relationships between LC9 and OAB. OR (solid lines) and 95% confidence levels (shaded areas) were adjusted for age, gender, education level, marital, PIR, race, smoking, drinking, hypertension, diabetes, and high cholesterol.

**Figure 3:**
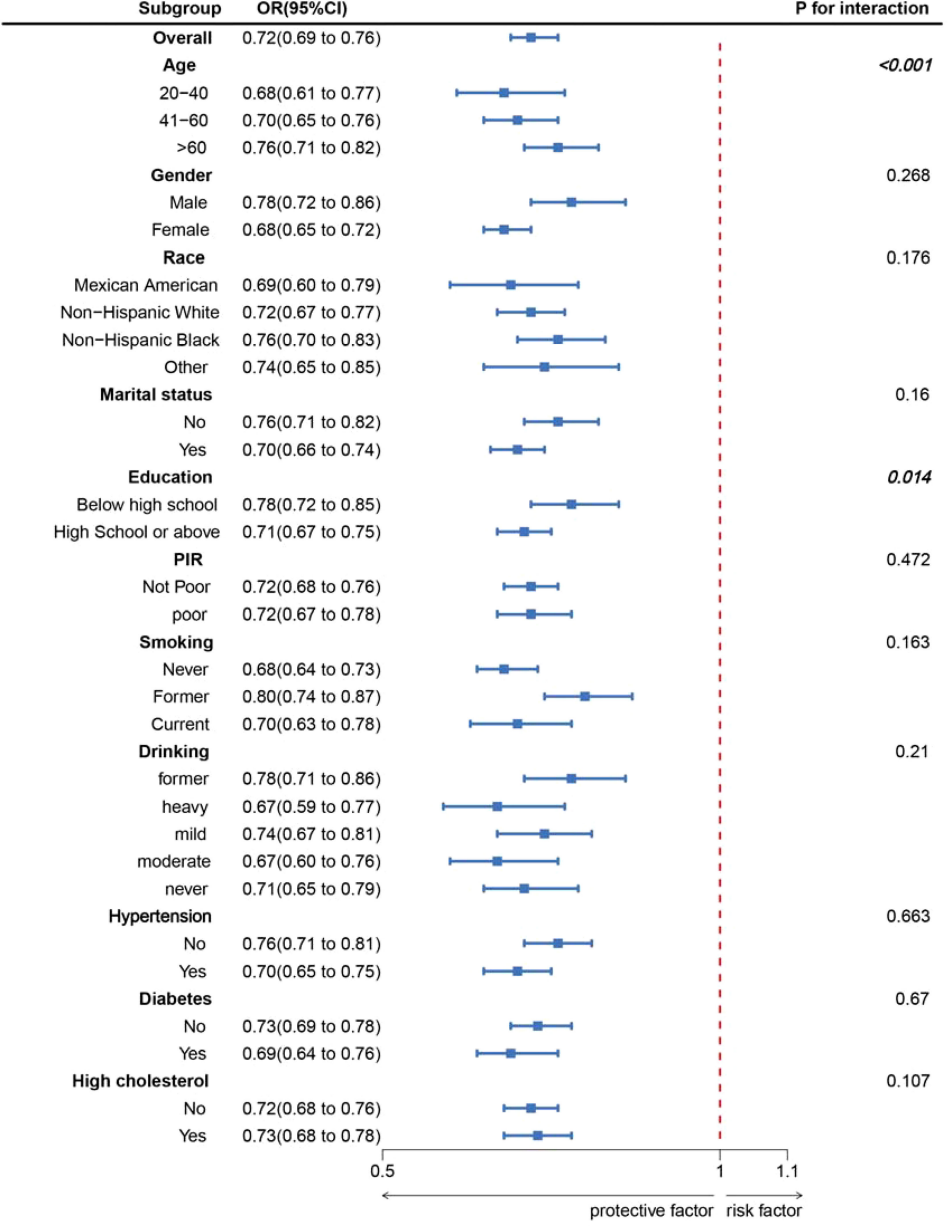
Subgroup analysis between LC9 and OAB. ORs were calculated as per 10-unit increase in LC9. Analyses were adjusted for age, gender, education level, marital, PIR, race, smoking, drinking, hypertension, diabetes, and high cholesterol.

As shown in **Figure 2**, LC9 was significantly and negatively associated with the probability of OAB (P for overall <0.001; P for nonlinear = 0.056). Subgroup analyses showed a constant association between LC9 and risk of OAB across all categories (**Figure 3**).

### AIP and prevalence of OAB

**Table 2** shows the relationship between OAB and AIP. In Model 3, after controlling for all variables, the third tertile (T3) was associated with a 15% increase in the odds of having OAB compared with the first tertile (T1) [OR=1.15 (1.00, 1.30)]. The positive correlation between OAB and AIP remained statistically significant when AIP was considered a continuous variable (OR=1.07, 95% CI: 1.01, 1.14). The results of Models 1 and 2 were equally consistent.

To ensure the stability of the results, this study performed multiple interpolations of missing data for LC9, AIP, and OAB, which remained consistent with the primary results (**Supplementary Table S5**).

### Association of LC9 and AIP

After accounting for every covariate, **Table 3** revealed a statistically significant correlation between LC9 and AIP (β=-0.27, 95% CI: -0.29, -0.26, P<0.001).

**Table 3:** Multivariate linear regression of LC9 and AIP.

	β	95%CI	P-value
LC9 - AIP	-0.27	(-0.29, -0.26)	<0.001

Adjusted for age, gender, education level, marital, PIR, race, smoking, drinking, hypertension, diabetes, and high cholesterol.

### Mediation effect

**Figure 4:**
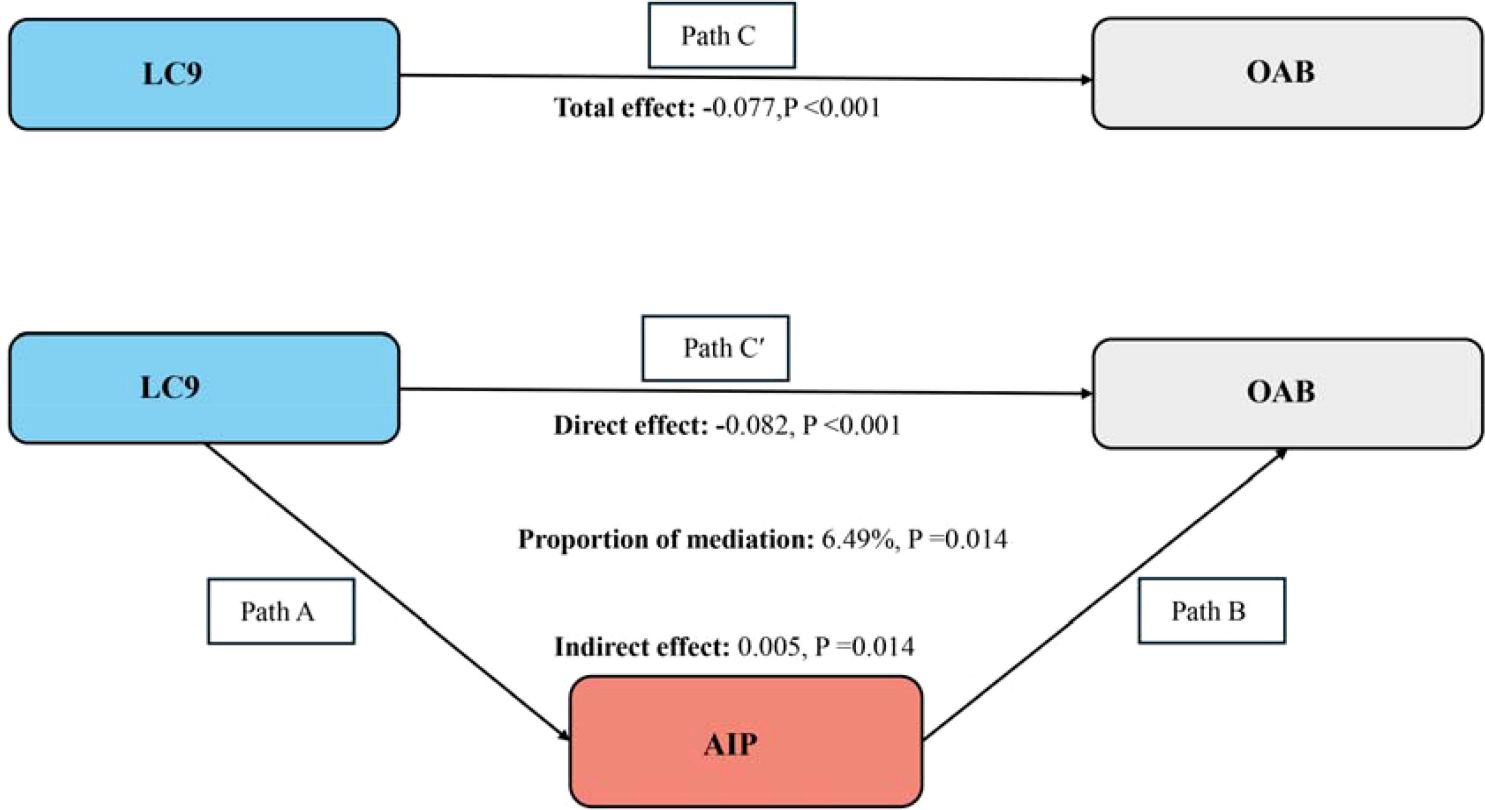
Schematic diagram of the mediation effect analysis. Path C indicates the total effect; path C′ indicates the direct effect. The indirect effect is estimated as the multiplication of paths A and B (path A*B). The mediated proportion is calculated as indirect effect/ (indirect effect + direct effect) × 100%. LC9, Life’s Crucial 9; OAB, overactive bladder; AIP, Atherogenic Index of plasma. Analyses were adjusted for age, gender, education level, marital, PIR, race, smoking, drinking, hypertension, diabetes, and high cholesterol.

The aforementioned analysis shows that our study complies with the prerequisites for conducting a mediation analysis. After correcting every variable, we observed the mediation effect of AIP (**Figure 4**). AIP (indirect impact = 0.005, P=0.014; direct effect = -0.082, P<0.001) mediated the relationship between LC9 and OAB risk to 6.49% (mediation proportion = indirect effect/(indirect effect + direct effect) *100%, P=0.014). As a result, it is feasible to see AIP as a mediating element in the relationship between LC9 and the probability of OAB.

## Discussion

In a nationally representative study of American adults, a significant association between LC9 and AIP, and OAB was observed. In the fully adjusted model, every 10-unit increase in LC9 was associated with a 0.27-fold reduction in AIP levels and a 28% reduction in OAB prevalence. For every unit increase in AIP, the prevalence of OAB increased by 7%. Mediated analysis showed that AIP partially mediated the relationship between LC9 and OAB, suggesting that LC9 may affect the occurrence of OAB by reducing lipid levels.

Life’s Essential 8 (LE8) is a measure of cardiovascular health, and previous research has established an association between LE8 and OAB (14). Our study not only confirms this association but also explores the relationship between Life’s Crucial 9 (LC9) and OAB, highlighting the significance of mental health in cardiovascular health. Mental health encompasses various aspects, including depression, anxiety, chronic stress, and traumatic stress, all of which are associated with an increased risk of cardiovascular disease. A recent study published in a JACC journal by Columbia University revealed that mental health issues are independently linked to increased cardiovascular disease risk (17), making it meaningful to incorporate mental health into cardiovascular health management. Importantly, we introduced the Atherogenic Index of Plasma (AIP) as a potential mediating factor. This finding supports the hypothesis that cardiovascular health may influence OAB risk by affecting lipid metabolism, thereby deepening our understanding of this association.

Mental health is a crucial component in enhancing cardiovascular health, and psychological factors such as depression (18) and anxiety (19) have been shown to have significant associations with overactive bladder (OAB) symptoms. OAB can affect mental health through various direct and indirect mechanisms, increasing the risk of depression. Among these mechanisms, the bladder-brain-gut axis has been innovatively proposed to elucidate their complex relationship (20), explaining how the brain regulates bladder function and how this communication can be disrupted, leading to the emergence of OAB symptoms. Psychological stress and emotional issues may also interfere with autonomic nervous system function (21), particularly affecting the balance between sympathetic and parasympathetic nervous systems, which may influence bladder storage capacity and urinary control, resulting in OAB symptoms. Furthermore, fluctuations in mental health status can lead to variations in serotonin levels within the body (22), subsequently affecting bladder function. An experimental study conducted on rats (23) revealed a close connection between serotonin levels in the central nervous system and urinary function. The findings indicate that when serotonin levels in the central nervous system decrease, two significant physiological responses occur: increased urinary frequency and overactivity of the detrusor muscle.

Lipid levels are a key component of Life’s Crucial 9 (LC9). The Atherogenic Index of Plasma (AIP), introduced by Dobiasova and Frohlich in 2001 (7), accurately reflects lipid metabolism, particularly the size of small dense low-density lipoprotein cholesterol (sdLDL-C) particles. It has been demonstrated that small dense LDL-C particles are highly sensitive to oxidative damage, leading to the development of atherosclerotic lesions (24). The AIP has been validated as a reliable indicator for predicting cardiovascular disease (CVD) (25, 26). Research has shown associations between AIP and urinary tract diseases such as the prevalence of kidney stones (27) and chronic kidney disease (28). Additionally, some studies indicate that dyslipidemia is a component of metabolic syndrome, with other factors of metabolic syndrome (such as hypertension, hyperglycemia, and abdominal obesity) potentially affecting bladder function either independently or in concert (29). However, there is currently a lack of research investigating the relationship between AIP and the incidence or progression of OAB, leading to a limited understanding of their association.

The four health behaviors—healthy diet, physical activity, smoking cessation, and good sleep—exert multifaceted effects on overactive bladder (OAB) through their interactions, collectively influencing the incidence and symptoms of OAB. A healthy diet can mitigate bladder irritation by reducing the intake of irritants such as coffee, carbonated beverages, and spicy foods. Additionally, adequate fiber consumption helps prevent constipation, minimizing its impact on pelvic nerve function and reducing pressure on the bladder (30). Regular physical activity not only strengthens pelvic floor muscles, enhancing bladder control (31) but also helps maintain a healthy weight, thereby alleviating abdominal pressure on the bladder (32). Smoking increases the excretion of nicotine in urine (33), a stimulant that can heighten bladder sensitivity, potentially leading to symptoms such as urgency and frequency (34). Smoking cessation can diminish nicotine’s irritative effects on the bladder, reducing the occurrence of urgency and frequency while also improving overall cardiovascular health, including blood supply to the bladder (35). Poor sleep or inadequate sleep quality directly affects bladder function and the incidence of nocturia. Studies have shown a significant association between sleep disturbances and lower urinary tract symptoms (LUTS) (36). Good sleep aids in regulating hormonal levels (37), which may enhance bladder function, while healthy sleep habits can further reduce nocturnal frequency. The combined effects of these health behaviors likely influence OAB incidence and symptom management through multiple pathways, including reducing bladder irritation, enhancing bladder control, improving circulation, and regulating hormonal levels.

Our study has several strengths. Firstly, we utilized robust data from the NHANES database, which benefits from a large sample size, standardized research protocols, strict quality control measures, and trained professionals for data collection and processing, ensuring the representativeness of the sample. We carefully adjusted for multiple covariates and conducted sensitivity analyses to enhance the reliability of our results, while also innovatively introducing the Atherogenic Index of Plasma (AIP) as a potential mediating factor, deepening our understanding of the association between Life’s Crucial 9 (LC9) and overactive bladder (OAB). Secondly, we relied on a scoring system for the diagnosis of OAB rather than self-reported measures, which helps reduce recall bias and subjective error.

However, it is important to note that our study has certain limitations. Firstly, the cross-sectional design of this research prevents us from establishing causal relationships between LC9 and OAB. Secondly, this study was based on a secondary analysis of NHANES data, focusing on U.S. adults. NHANES, as a national health and nutrition survey, provides large sample size and high-quality data, making it an essential resource for epidemiological research. However, since the study population is limited to the United States, the generalizability of our findings to other populations may be affected. Differences in LC9 levels, lifestyle behaviors, healthcare systems, and genetic backgrounds across countries may influence the association between LC9 and OAB. Therefore, future studies are needed in diverse populations to validate the generalizability of our findings.

Nevertheless, our study has important public health implications. First, it provides epidemiological evidence supporting the potential role of LC9 in OAB prevention and management, offering valuable insights for future prospective cohort studies, clinical interventions, and mechanistic research. Additionally, our methodology and findings may serve as a reference for studies in other countries, contributing to a broader understanding of how environmental and lifestyle factors impact OAB across different populations. Such international research efforts could help develop more effective global strategies for OAB prevention and management.

## Conclusion

In conclusion, we found a significant negative correlation between Life’s Crucial 9 (LC9) and overactive bladder (OAB), with the Atherogenic Index of Plasma (AIP) serving as a partial mediating factor in this relationship. This finding underscores the potential link between cardiovascular health and OAB, highlighting the importance of lipid metabolism in this context. Our study offers new insights into the prevention and management of OAB, emphasizing that a comprehensive approach to improving cardiovascular health and addressing lipid metabolism may contribute to a reduction in the prevalence of OAB.

## Data Availability

The datasets presented in this study can be found in online repositories. The names of the repository/repositories and accession number(s) can be found below: https://www.cdc.gov/nchs/nhanes/index.htm.
